# Social anxiety and life impact in adults who clutter and stutter: a comparative study with pure stuttering

**DOI:** 10.3389/fpsyg.2026.1810425

**Published:** 2026-05-28

**Authors:** Shuta Tomisato, Takanori Mori, Kazumi Asano, Yasuto Yada, Koichiro Wasano, Takeyuki Kono, Hiroyuki Ozawa

**Affiliations:** 1 Department of Otorhinolaryngology, Head and Neck Surgery, Keio University School of Medicine, Shinjuku, Tokyo, Japan; 2 Department of Language Sciences, Tokyo Metropolitan University, Hachioji, Tokyo, Japan; 3 Department of Otolaryngology, Head and Neck Surgery, Tokai University School of Medicine, Isehara, Kanagawa, Japan

**Keywords:** cluttering, stuttering, fluency disorders, social anxiety disorder, speech disorders

## Abstract

Social anxiety disorder (SAD) is frequently reported to coexist with stuttering; however, its occurrence in the context of cluttering remains largely unexplored. This study determined whether SAD co-occurs in individuals with disfluency after differentiating those with cluttering with stuttering from those with stuttering alone. Forty participants aged ≥18 years with a chief complaint of stuttering-like disfluencies (SDF) were included. Based on established criteria, participants were categorized into two groups: cluttering with stuttering (*n* = 14) and stuttering alone (*n* = 26). All participants completed Japanese versions of the following: Modified Erickson Communication Attitude Scale (S-24-J), Liebowitz Social Anxiety Scale (LSAS-J), Brief Unhelpful Thoughts and Beliefs About Stuttering Scale (UTBAS-6-J), and Overall Assessment of the Speaker’s Experience of Stuttering for Adults (OASES-A-J). Although mean scores on the LSAS-J and other questionnaires did not differ significantly between the groups, correlational analyses revealed distinct psychological profiles. A significant correlation between SDF frequency and OASES-A-J Section 1 scores was observed only in the stuttering-alone group. These findings indicate that while SAD can co-occur in both groups, the relationships between objective severity, communication passivity, and nonadaptive cognition differ. This underscores the distinct clinical natures of cluttering with stuttering and pure stuttering, highlighting the need for a nuanced, tailored approach to their assessment and clinical management.

## 1 Introduction

Cluttering and stuttering are distinct disorders, both characterized by language disfluency. Cluttering manifests primarily as speech uttered at a fast articulation rate accompanied by disorganized word order and sentence structure (Daly and Burnett, 1999; Van Zaalen-Op’t Hof and Reichel, 2015). By contrast, stuttering manifests primarily as speech uttered in repetitions, prolongations, stretching out of sounds, and blocks (Yairi and Seery, 2025). Although these two disorders are distinct, they are not mutually exclusive; stuttering-like symptoms can also occur in cases of cluttering. Thus, patients presenting with a chief complaint of speech disfluency may have a mixture of stuttering and cluttering, making accurate diagnosis paramount.

Research has indicated that between 12 and 43% of individuals diagnosed with speech disfluency also exhibit symptoms consistent with cluttering (Van Riper, 1982; van Zaalen-Op’t Hof et al., 2009; Ward, 2011). For example, Ward (2018) reports that about 25% of English-speaking patients diagnosed with stuttering exhibit symptoms of cluttering. A similar phenomenon has been observed among Japanese speakers; 15–38% of individuals diagnosed with disfluent speech have also been identified as exhibiting cluttering tendencies (Miyamoto et al., 2007; Tomisato et al., 2024a). Clinically, distinguishing between cluttering and stuttering is important for the patient, since it is reasonable to assume that treatment will be different for each disorder (Ward, 2011; Van Zaalen-Op’t Hof and Reichel, 2015; St. Louis et al., 2018).

Another confounding issue is that cluttering lacks a definitive definition (Miyamoto, 2011). This has led to a situation in which some studies have inadvertently included samples comprising a combination of stutterers and clutterers, which, in turn, has resulted in the publication of mixed findings and uncertain approaches to effective treatments. Thus, speech-language pathologists, researchers, and clinicians would benefit greatly by having clear, differentiating characteristics and symptoms of cluttering and stuttering.

One common way to identify cluttering is through the least common denominator (LCD) approach, which focuses on core characteristics that are consistently present in individuals that clutter, rather than on a wide range of symptoms. The LCD defines cluttering as a fluency disorder wherein segments of a conversation in the speaker’s native language are typically perceived as being too fast overall, too irregular, or both (St. Louis et al., 2007). However, this definition is subjective, because the listener (often a speech therapist or clinician) is the one who judges whether the speech is too fast overall, too irregular, or both. Furthermore, this LCD approach does not delineate clear threshold values for each of these characteristics.

Another way employs a quantitative approach. To diagnose cluttering or cluttering-stuttering (a mixture of cluttering and stuttering), van Zaalen-Op’t Hof et al. (2009) calculated a ratio of disfluency (RDF) by dividing the frequency of normal disfluency (NDF) by the frequency of stuttering-like disfluency (SDF). An RDF of ≥1 is diagnostic of cluttering. However, with Japanese speakers, using this RDF criterion alone has led to an overdiagnosis of cluttering (Iimura and Miyamoto, 2021). Therefore, a quantitative LCD approach specific for the unique characteristics of the Japanese language is needed to more accurately discriminate between true clutterers and stutterers.

Our previous study adjusted the RDF threshold criterion higher (i.e., >1) for native-Japanese speakers, and used this RDF criterion and the mean articulation rate (MAR) to identify cluttering (Tomisato et al., 2024a). We reported that using an RDF > 1.2 and a MAR > 7.5 morae/s were useful for identifying cluttering (Tomisato et al., 2024a). In the present study, we used this method to differentiate cluttering from stuttering.

Many studies have found that social anxiety disorder (SAD) and stuttering often co-occur. Individuals who stutter have a six- to sevenfold increased likelihood of experiencing SAD compared to those who do not stutter (Iverach et al., 2009). Moreover, 40% of adults with stuttering who seek treatment are also diagnosed with SAD (Blumgart et al., 2010). Unfortunately, these studies did not differentiate between stuttering and cluttering, so it is unclear whether both disorders are associated with SAD.

As with stuttering, cluttering impairs verbal fluency. Questionnaire-based surveys suggest that people who clutter tend to have higher levels of depressive symptoms and psychosomatic symptoms, which contribute to a decline in their well-being (Icht et al., 2023; Zukerman et al., 2024). Just as with stuttering, it is thought that cluttering may be associated with SAD, but there are scarce studies that have directly investigated the relationship between cluttering and SAD.

There is no correlation between the objective severity of stuttering and the subjective severity of stuttering, as measured by questionnaires (Mulcahy et al., 2008; Manning and Gayle Beck, 2013; Iverach et al., 2018; Tomisato et al., 2022). For instance, the scores of objective severity indices (e.g., Percent Syllables Stuttered [%SS] and Stuttering Severity Instrument) were not significantly related to results from an anxiety questionnaire or scores on the Overall Assessment of the Speaker’s Experience of Stuttering (OASES; Manning and Gayle Beck, 2013). This finding is consistent with reports on Japanese speakers (Tomisato et al., 2022) and adolescents (Mulcahy et al., 2008). However, some reports have indicated a significant correlation between %SS and scores on the Social Anxiety Questionnaire (Lei et al., 2024), as well as between %SS and scores on Section 1 (General Information) of the OASES (Blumgart et al., 2012). Nonetheless, the correlation coefficients in these cases were consistently weak. These findings suggest the absence or weak nonsignificant correlations between objective severity of stuttering and subjective severity measured using a questionnaire. Conversely, many studies have demonstrated a robust correlation between the scores on these two questionnaires (Mulcahy et al., 2008; Manning and Gayle Beck, 2013; Sakai et al., 2017; Ward et al., 2021; Tomisato et al., 2022; Lei et al., 2024). Again, these studies did not differentiate between cluttering and stuttering patients. Examining these two conditions separately could potentially disentangle the relationship between objective severity and subjective severity as measured by using questionnaires.

In the present study, we investigated several aspects in participants who presented with stuttering-like disfluency (SDF) as their chief complaint. A key assessment procedure we performed before SAD assessment was to objectively group participants first into two different groups: (1) a group that included only those who have stuttering with cluttering, and (2) a group that has participants who have only stuttering. Our first objective was to determine whether the previously reported association between social anxiety and stuttering are similarly observed in patients who have cluttering. Our second objective was to determine whether objective severity of the disfluency and questionnaire scores are correlated for the cluttering with stuttering group (Cl-St-group) and the stuttering group (St-group). We used the same methodology used for differential diagnosis of cluttering and stuttering that was used in a previous study (Tomisato et al., 2024a). This 2024 study focused on differentiating between stuttering and cluttering in adults who presented at the hospital with the primary complaint of language disfluency. The results of each questionnaire were compared between and within the two groups.

## 2 Methods

### 2.1 Participants and groups

Participants were recruited between July 2020 and February 2024. We included 40 patients who visited the Department of Otorhinolaryngology-Head and Neck Surgery at Keio University Hospital. All of them had a chief complaint of SDF and met the following criteria: aged ≥18 years, had no previous specialized treatment, and had a SDF frequency of ≥1.0, as described in the next paragraph (i.e., STS). Verbal consent was obtained, and participants were allowed to opt-out of the study at any time without prejudice. The study was approved by the Ethics Committee of Keio University School of Medicine.

On their first or second visit, participants were administered the Standardized Test for Stuttering (STS; Ozawa et al., 2016), an objective test for stuttering in Japanese speakers. Similar to the Stuttering Severity Instrument (SSI-4; Riley, 2009), the STS comprises a variety of tasks that results in an estimate of stuttering frequency. The participants were assigned to either a stuttering group or cluttering with stuttering group based on data obtained from the participants’ STS monolog, using the method described in our previous study (Tomisato et al., 2024a). First, the frequencies of SDF and NDF were calculated using audio data from each participant. The disfluencies in SDF included “tense word repetition,” “tense part-word repetition,” “prolongation,” and “block,” while those in NDF included “word repetition,” “part-word repetition,” “interjection,” “revision,” and “phrase repetition” (van Zaalen-Op’t Hof et al., 2009). The frequency was calculated by dividing the number of occurrences of SDF or NDF by the number of “bunsetsu” rather than the number of words. “Bunsetsu” is a linguistic unit in the Japanese language that is longer than a word but shorter than a phrase. As stuttering occurs at the beginning of “bunsetsu,” it is common to use the “bunsetsu” unit as the denominator when calculating the frequency of stuttering in Japanese speakers (LaSalle and Huffman, 2015; Ozawa et al., 2016; Iimura and Miyamoto, 2021).

Next, MAR was calculated from audio data (speech-only recordings) obtained during each participant’s STS monolog. This was done by dividing the number of pronounced morae by the time required to pronounce them. The following three conditions in the speech data had to be satisfied to obtain a valid MAR: (1) presence of 8–20 morae, (2) absence of SDF, and (3) ≥ 250 msec of speech without pauses (Tomisato et al., 2024a). The MAR was the mean value of three points randomly selected from the audiometric data of each participants’ monolog.

Participants with an RDF (NDF/SDF) of >1.2 and a MAR of >7.5 were assigned to the group of cluttering with stuttering (Cl-St-group); the remaining participants were assigned to the group of stuttering (St-group). To assess the reproducibility of our group designations, two independent evaluators assessed the audio data of all participants. The degree of agreement of the two evaluators’ assessments was tested using Cohen’s kappa coefficient (Landis and Koch, 1977). The calculated kappa coefficient was 0.73. The two evaluators initially made different diagnoses for five participants. Ultimately, two of these participants were diagnosed with cluttering with stuttering and three with stuttering, based on the average value of the diagnoses by the two evaluators. In the end, 14 participants were assigned to the Cl-St-group, and 26 participants were assigned to the St-group.

The male-to-female ratio in the Cl-St-group (13:1) and St-group (20:6) groups was not significantly different (Fisher’s exact test, *p* = 0.38; Table 1), nor was their mean age (Cl-St-group: mean ± standard deviation [SD], 25.1 ± 7.4 years; St-group: 25.7 ± 6.9 years; t-test, *p* = 0.40; Table 1). One patient in the Cl-St-group was additionally diagnosed with attention deficit hyperactivity disorder (ADHD), and one patient in the St-group was diagnosed with both ADHD and autism spectrum disorder.

**Table 1:** Summary of participant demographics.

	Cl-St-group	St-group	*p* value
N	14	26	
Sex ratio male:female	13:1	20:6	0.38^a^
Age (years), Mean ± SD	25.1 ± 7.4	25.7 ± 6.9	0.40^b^
SDF Mean ± SD	7.3 ± 5.4	19.2 ± 22.2	–
NDF Mean ±SD	18.7 ± 7.8	21.0 ± 17.5	–
RDF Mean ±SD	4.8 ± 6.2	2.6 ± 3.0	–
MAR (morae /s) Mean ±SD	8.7 ± 1.1	6.7 ± 1.0	–

The differential diagnosis between cluttering with stuttering (Cl-St-group) and stuttering (St-group) was made based on findings of previous studies. Of the patients who presented with the chief complaint of language disfluency, 14 (35%) were diagnosed with cluttering using the criteria herein. SDF: frequency of stuttering-like disfluency (per 100 “bunsetsu”); NDF: frequency of normal disfluency (per 100 “bunsetsu”); RDF: ratio of disfluencies (NDF / SDF); MAR: mean articulation rate (morae/sec). Statistical tests were not performed for SDF, NDF, RDF, and MAR, as these variables were used for group assignment.

a Fisher’s exact test.

b t-test.

### 2.2 Test evaluation

The following four tests were completed by participants during their first visit to our department: (1) Modified Erickson Communication Attitude Scale - Japanese version (S-24-J; Sasanuma and Ito, 1998; Iimura and Ishida, 2022); (2) Liebowitz Social Anxiety Scale - Japanese version (LSAS-J; Liebowitz, 1987; Asakura et al., 2002); (3) Brief Unhelpful Thoughts and Beliefs About Stuttering Scale - Japanese version (UTBAS-6-J; St Clare et al., 2009; Iverach et al., 2011, 2016; Tomisato et al., 2024b); and (4) Overall Assessment of the Speaker’s Experience of Stuttering for Adults – Japanese version (OASES-A-J; Sakai et al., 2017). All the tests were in self-report, questionnaire format.

The S-24-J measures respondents’ attitudes toward communicating, specifically with regard to stuttering; it consists of 24 “yes”–“no” questions (Sasanuma and Ito, 1998; Iimura and Ishida, 2022). Answers that are characteristic of negative attitudes toward communication are given one point. The S-24-J score is calculated by summing the total number of points. The higher the score, the more negative attitude toward communication the person has.

The LSAS-J is a 24-item screening tool for SAD (Liebowitz, 1987; Asakura et al., 2002). Respondents are asked to rate their “fear” and “avoidance” of 24 different situations on a four-point scale (0 = none; 3 = severe). The total LSAS-J score is calculated by summing the “fear” and “avoidance” scores. The higher the score, the stronger the person’s tendency is toward social anxiety.

The UTBAS-6-J is a shortened Japanese version of the UTBAS; it is a six-item scale that quantifies the degree of non-adaptive cognition in individuals who stutter (St Clare et al., 2009; Iverach et al., 2011; Chu et al., 2017; Tomisato et al., 2024b). For each of the six items of the UTBAS-6-J, respondents are asked to answer three questions on a five-point scale (1 = never or not at all; 5 = always or totally): section 1, how frequently I have these thoughts; section 2, how much I believe these thoughts; and section 3, how anxious these thoughts make me feel. Subsection scores are obtained by summing the scores for each of the three questions (i.e., sections 1–3). The overall score is obtained by summing the three subsection scores. Higher scores indicate non-adaptive, greater frequency of negative and unhelpful thoughts and beliefs related to their stuttering.

The OASES-A-J is the adult version of the OASES (Sakai et al., 2017). It is a questionnaire designed to evaluate the experience of stuttering from the perspective of the individual who stutters (Yaruss and Quesal, 2006). The OASES-A-J comprises four sections, each having 25 items: (1) general perspectives about stuttering; (2) affective, behavioral, and cognitive reactions to stuttering; (3) functional communication difficulties; and (4) impact of stuttering on the speaker’s quality of life. Respondents are asked to rate each of items within the above four sections on a five-point scale. For each of the four sections, a score is obtained by summing the ratings for each response within that particular section. The overall OASES-A-J score is obtained by calculating the mean of the four section scores. Higher scores indicate a more negative experience related to the individual’s stuttering.

The test scores of participants from Cl-St-group and St-group were compared using unpaired t-tests. Within-group correlations were calculated to determine associations between the frequency of SDF and test scores. All statistical analyses were conducted using SPSS 26 software (IBM, NY, United States).

## 3 Results

### 3.1 Lack of group differences in overall test battery performance

The mean scores and effect sizes (Cohen’s d) for the two groups are presented in Table 2. T-test results revealed no statistically significant differences between the two groups across all measures or their subsections. However, while *p*-values remained above the significance threshold, moderate effect sizes were observed in certain components, such as the LSAS-J avoidance subscale (Table 2).

**Table 2:** Test performance of cluttering with stuttering group and stuttering group.

Questionnaire	Cl-St-group (*n* = 14) Mean ±SD	St-group (*n* = 26) Mean ±SD	*p*-value^a^	Effect size (Cohen’s d)
S-24-J	19.1 ± 2.9	19.3 ± 2.6	0.83	0.07
**LSAS-J**	51.8 ± 25.5	59.0 ± 29.2	0.44	0.26
Fear	30.4 ± 13.9	32.2 ± 13.7	0.70	0.13
Avoidance	21.3 ± 12.1	26.8 ± 16.2	0.28	0.36
**UTBAS-6-J**	47.8 ± 13.7	50.6 ± 18.2	0.62	0.17
1: How frequently I have these thoughts	16.9 ± 4.6	17.7 ± 6.4	0.71	0.12
2: How much I believe these thoughts	13.9 ± 5.1	14.6 ± 6.7	0.76	0.10
3: How anxious these thoughts make me feel	17.6 ± 5.8	18.4 ± 6.6	0.73	0.12
**OASES-A-J**	3.27 ± 0.51	3.29 ± 0.57	0.91	0.04
1: General perspectives about stuttering	3.78 ± 0.55	3.23 ± 0.60	0.47	0.24
2: Affective, behavioral, and cognitive reactions to stuttering	3.53 ± 0.67	3.41 ± 0.63	0.57	0.19
3: Communication in daily situations (Functional communication difficulties)	2.87 ± 0.55	3.13 ± 0.68	0.24	0.39
4: Impact of stuttering on the speaker’s quality of life	3.24 ± 0.65	3.32 ± 0.81	0.76	0.10

Cl-St-group, subjects with cluttering with stuttering; St-group, subjects with stuttering; SD, standard deviation; S-24-J, Modified Erickson Communication Attitude Scale - Japanese version; LSAS-J, Liebowitz Social Anxiety Scale - Japanese version; UTBAS-6-J, Brief Unhelpful Thoughts and Beliefs About Stuttering Scale - Japanese version; OASES-A-J, Overall Assessment of Speaker’s Experience of Stuttering for Adults – Japanese version.

a *p*-value for t-test comparing cluttering and stuttering groups.

### 3.2 Correlations between questionnaires

Pairwise correlations of within-group performance between tests were examined next (Table 3). We found characteristic correlations between performance on the S-24-J and performance on the other tests (e.g., Tomisato et al., 2024b). In the Cl-St-group, for example, we identified a significant correlation between S-24-J and UTBAS-6-J performance (Pearson’s *r* = 0.60, *p* = 0.02), as well as with UTBAS-6-J and LSAS-J performance (Pearson’s *r* = 0.63, *p* = 0.02). Conversely, in the St-group, we found significant correlations between S-24-J and LSAS-J performance (Pearson’s *r* = 0.68, *p* < 0.001) and between S-24-J and OASES-A-J performance (Pearson’s *r* = 0.44, *p* = 0.03), in addition to UTBAS-6-J and LSAS-J performance (Pearson’s *r* = 0.45, *p* = 0.02).

**Table 3:** Within-group, pairwise correlation coefficients (r) for test battery.

Questionnaire	S-24-J		LSAS-J		UTBAS-6-J		OASES-A-J	
	Cl-St	St	Cl-St	St	Cl-St	St	Cl-St	St
S-24-J	1.0	1.0	0.34	0.68**	0.60*	0.34	0.19	0.44*
LSAS-J	0.34	0.68**	1.0	1.0	0.63*	0.45*	0.60*	0.66**
UTBAS-6-J	0.60*	0.34	0.63*	0.45*	1.0	1.0	0.77**	0.78**
OASES-A-J	0.19	0.44*	0.60*	0.66**	0.77**	0.78**	1.0	1.0

Cl-St, subjects with cluttering with stuttering; St, subjects with stuttering; same abbreviations as in Table 2. *Pearson correlation coefficients; *p < 0.05, **p < 0.01.

### 3.3 Correlation between the frequency of SDF and questionnaire scores

**Figure 1:**
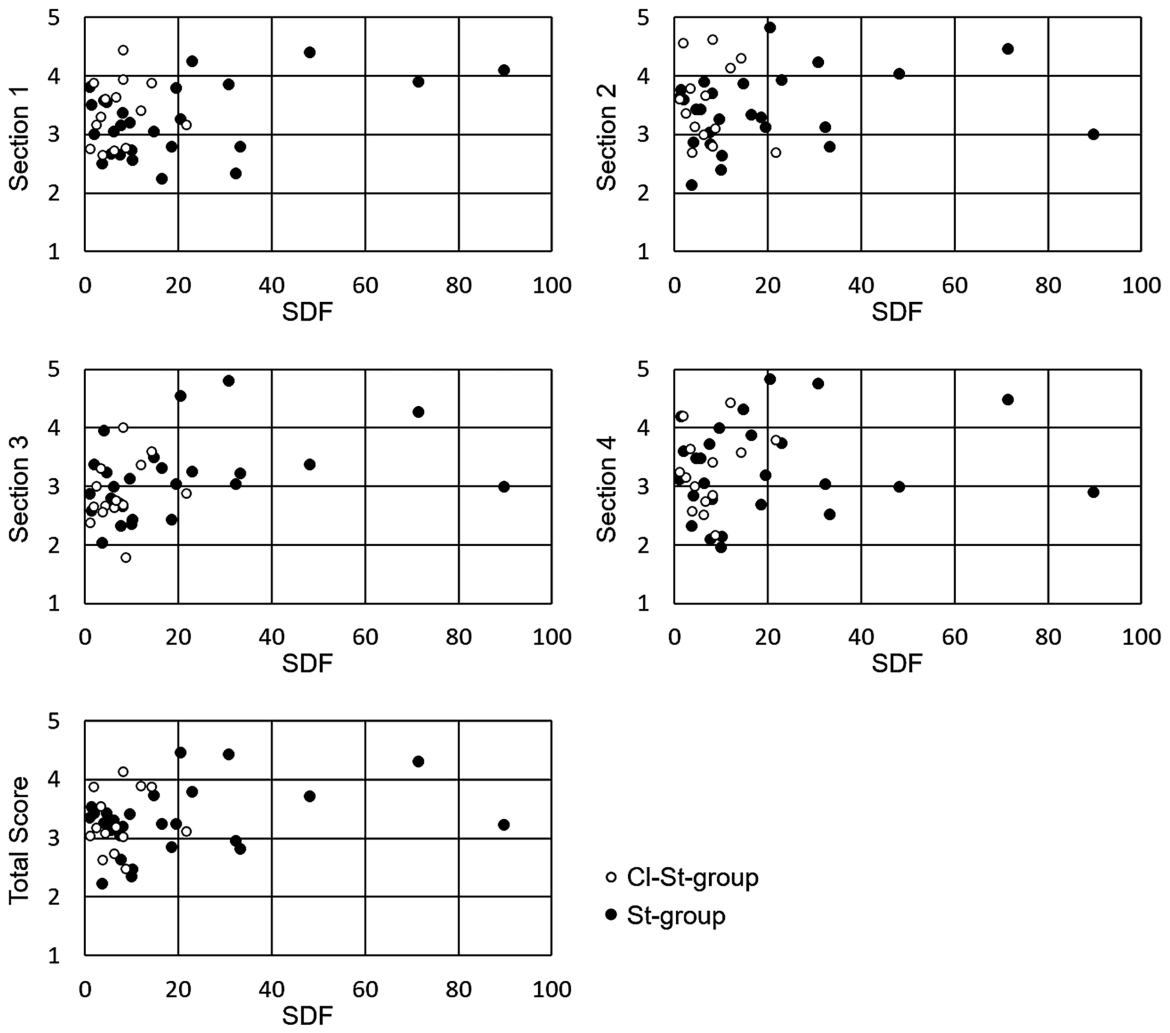
Correlations between SDF frequency and OASES-A-J scores. Only OASES-A-J Section 1 scores and frequency of SDF showed a significant correlation in the stuttering group. Cl-St-group, subjects with cluttering with stuttering; St-group, subjects with stuttering; SDF, stuttering-like disfluencies; OASES-A-J, Overall Assessment of the Speaker’s Experience of Stuttering for Adults – Japanese version.

We assessed pairwise correlations between the frequency of SDF in speech features and test scores of the Cl-St-group and the St-group (Table 4). Of particular interest for differentiating cluttering from stuttering were the SDF and OASES-A-J Section 1 scores. In the Cl-St-group, SDF and OASES-A-J Section 1 scores were not significantly correlated (Pearson’s *r* = 0.15, *p* = 0.61). By contrast, in the stuttering group, SDF and OASES-A-J Section 1 scores were significantly correlated (Pearson’s *r* = 0.44, *p* = 0.02; Figure 1).

**Table 4:** Pearson’s correlation coefficients (r) for correlation between the frequency of SDF and questionnaire scores.

Questionnaire	SDF	
	Cl-St	St
S-24-J	0.38	−0.12
**LSAS-J**	0.07	0.03
Fear	0.12	0.01
Avoidance	0.007	0.05
**UTBAS-6-J**	0.29	0.12
1: How frequently I have these thoughts	0.05	0.12
2: How much I believe these thoughts	0.38	0.24
3: How anxious these thoughts make me feel	0.21	−0.03
**OASES-A-J**	0.14	0.32
1: General perspectives about stuttering	0.15	0.44*
2: Affective, behavioral, and cognitive reactions to stuttering	−0.16	0.22
3: Communication in daily situations (Functional communication difficulties)	0.27	0.35
4: Impact of stuttering on the speaker’s quality of life	0.25	0.13

SDF, Stuttering-like disfluencies; same abbreviations as in previous tables. *p < 0.05. Cl-St, subjects with cluttering with stuttering; St, subjects with stuttering.

## 4 Discussion

Cluttering and stuttering are often referred to interchangeably in clinical settings, partly because knowledge about their distinctive symptoms is lacking. The present study aimed to identify and clarify the clinical characteristics that distinguish cluttering from stuttering by using a variety of self-report tests that assess communication attitudes and SAD. We also sought to determine whether SAD co-occurs only with stuttering or also co-occurs with cluttering, and whether the severity of cluttering and stuttering is correlated with the severity of SAD. A comparative analysis of test scores revealed no significant differences between the cluttering and stuttering groups. For example, the mean S-24-J score for the Cl-St-group was 19.1 (±2.9), while the mean S-24-J score for St-group was 19.3 (±2.6). This indicates that communication styles (i.e., passivity) of persons with cluttering with stuttering and those with stuttering are comparable (Table 2). This comparability pattern was also true for the total scores on the UTBAS-6-J (Cl-St-group mean = 47.8 ± 13.7, St-group mean = 50.6 ± 18.2) and the OASES-A-J (Cl-St-group mean = 3.27 ± 0.51; St-group mean = 3.29 ± 0.57). Participants having cluttering with stuttering and those having only stuttering were also similar in terms of maladaptive cognition and the impact of their disfluency on their overall lives (Table 2).

Importantly, scores on the LSAS-J, a screening tool for SAD, indicated that the two groups in the present study were comparable in terms of SAD co-occurrence. This extends the findings of previous studies that stuttering frequently co-occurs with SAD (Iverach et al., 2009; Blumgart et al., 2010; Chu et al., 2020; Tomisato et al., 2022). Previous research on cluttering has shown that cluttering is associated with high levels of depressive symptoms and low levels of well-being (Icht et al., 2023; Zukerman et al., 2024). In view of the established links between social anxiety and depression (Stein et al., 2001), the present findings imply that social anxiety may be a contributing factor to the observed associations between cluttering and depression. Similarly, the reported correlation of cluttering with diminished well-being (Russell and Topham, 2012; De Castella et al., 2014) supports this hypothesis. The results further underscore the need for clinicians to take into consideration the possible presence of SAD in managing and treating patients with cluttering.

Our correlational analysis assessing the relationships between scores on the different tests also showed that the two groups differed. In the Cl-St-group, communication passivity (S-24-J) and nonadaptive cognition (total score of UTBAS-6-J) were significantly correlated (Pearson’s *r* = 0.60; Table 3). In the St-group, communication passivity (S-24-J) and social anxiety (total score of LSAS-J) were significantly correlated (*r* = 0.68; Table 3), as were communication passivity (S-24-J) and impact on overall quality of life (total score of OASES-A-J), (*r* = 0.44; Table 3). In our previous study—which had a wholly distinct group of participants from those in the present study—a strong correlation was identified between the scores of the S-24-J and the LSAS-J in the stuttering group (Tomisato et al., 2022). In Sakai et al.’s study, a strong correlation was observed between the total scores of the S-24-J and the OASES-A-J in the stuttering group. Our previous study was conducted prior to our implementation of the differential diagnosis between cluttering and stuttering. Sakai et al.’s study focused on individuals who self-reported stuttering. Given that cluttering symptoms are estimated to coexist in 12 to 43% of cases of speech disfluency (Van Riper, 1982; van Zaalen-Op’t Hof et al., 2009; Ward, 2011; Ward, 2018), it is highly probable that both samples included a mix of pure stuttering and cluttering with stuttering. In the present study, significant associations between communication passivity (S-24-J) and social anxiety (LSAS-J) or impact on quality of life (OASES-A-J) were primarily observed within the pure stuttering group. By explicitly differentiating between pure stuttering and cluttering with stuttering, this study provides a clearer understanding of the distinct psychological and cognitive profiles associated with each condition.

While the precise explanations for these differences remain elusive, one possibility is that they could be related to the distinct nature of cluttering and stuttering. We hypothesize that they are distinct disorders. Stuttering is characterized by SDF. Cluttering, on the other hand, is characterized not only by SDF, but also by NDF, articulation rate, word order, and sentence structure disfluency (Daly and Burnett, 1999; Van Zaalen-Op’t Hof and Reichel, 2015). From a clinical perspective, our findings suggest that the psychological mechanisms underlying communication passivity differ between these groups. In the St-group, the strong correlation between communication passivity and social anxiety—consistent with previous findings by Sakai et al. (2017) and Tomisato et al. (2022)—reaffirms that for pure stutterers, passivity is closely tied to the fear of social evaluation. Conversely, in the Cl-St-group, the correlation between communication passivity and nonadaptive cognition (UTBAS-6-J) suggests that their passivity may be more closely associated with maladaptive thought patterns regarding their speech production itself. This differentiation is crucial for selecting the most efficacious treatment approach for each group.

The efficacy of cognitive-behavioral therapy (CBT) in treating stuttering is well-documented (Menzies et al., 2009; Mongia et al., 2019; Reddy et al., 2010), particularly in addressing associated social anxiety and maladaptive cognition (Iverach et al., 2017). Our results suggest that while CBT may be an efficacious treatment for both disorders, the primary therapeutic targets might differ. For pure stuttering, interventions focusing on social anxiety and desensitization remain paramount. However, for cluttering with stuttering, given its significant correlation with nonadaptive cognition identified in this study, clinical management should perhaps prioritize cognitive restructuring of the patient’s beliefs about their speech control and fluency. While only one study has examined CBT for cluttering (Langevin and Boberg, 1996), its effectiveness may be maximized by tailoring the cognitive components to the specific disfluency profile of the individual. More research is needed to determine the optimal, tailored CBT protocols for these two distinct speech disfluencies.

In the present study, we identified a significant correlation between the frequency of SDF and scores on Section 1 of the OASES-A-J in the St-group (*r* = 0.44) but not in the Cl-St-group (*r* = 0.15). This finding is similar to that of a previous study that found a weak but significant correlation between stuttering frequency and OASES-A Section 1 scores (*r* = 0.23; Blumgart et al., 2012). Although there is no prior research on the cluttering of this correlation, Blumgart and colleagues’ results were considered to be specific to stuttering. This underscores the necessity for distinguishing cluttering from stuttering in order to more clearly identify the clinical characteristics of stuttering and treat it appropriately. Furthermore, when we analyzed our data with scatter plots of SDF and OASES-A-J scores, data points of the Cl-St-group were predominantly concentrated in the upper-left area of the graph (Figure 1), an area characterized by a low frequency of SDF and high OASES-A-J scores. Given the equivalence of the OASES-A-J scores (Table 2), it can be deduced that while the impact of disfluency on daily life was comparable, the objective severity calculated using SDF was lower in the Cl-St-group (Table 1). In clinical settings, for groups in which stuttering and cluttering coexist, SDF is typically treated as the primary objective measure of disfluency severity. However, the correlation mentioned above suggests that SDF alone may not be a sufficient objective severity measure for individuals with co-occurring cluttering. Although the Cl-St-group exhibited a lower frequency of SDF compared to the St-group, their subjective impact (OASES-A-J) remained high, likely due to the additional burden of cluttering-related symptoms (e.g., high NDF and MAR as shown in Table 1). This indicates the necessity for a severity assessment for cluttering that is distinct from that used for stuttering. More research is needed to bolster this hypothesis.

### 4.1 Strengths and limitations

In summary, the present study showed that generally, while the cluttering and stuttering groups scored comparably on tests assessing communication and SAD, they differed in correlational analysis assessing the relationships between scores on the different tests. In the cluttering group, communication passivity and nonadaptive cognition were significantly correlated, whereas in the stuttering group, communication passivity and social anxiety were significantly correlated, as were communication passivity and impact on overall quality of life. Nonetheless, this study has some limitations.

First, the study’s statistical power was limited by the relatively small sample sizes (*n* = 14 for the Cl-St-group and *n* = 26 for the St-group). Consequently, some comparisons—such as those in the LSAS-J avoidance and OASES-A-J subsections—exhibited moderate effect sizes (Table 2) but did not reach statistical significance. These results suggest that clinically meaningful differences may exist that would potentially be confirmed in a larger sample. Similarly, observed differences in correlational patterns between groups should be treated as exploratory; a lack of significance in one group does not definitively prove a statistical difference between the two correlation coefficients. These associations remain tentative and require further verification with a larger dataset.

Second, the cross-sectional design of this study precludes any definitive causal interpretations. While significant associations were found—such as the correlation between the S-24-J and UTBAS-6-J in the Cl-St-group—they do not establish temporal precedence or a directional causal mechanism. These relationships should be considered hypothetical until confirmed by longitudinal or intervention studies. Furthermore, the findings may be influenced by self-selection bias, as the participants were individuals seeking clinical support at a university hospital. This sample may represent individuals with more pronounced symptoms than the general population of those who clutter or stutter. Therefore, while these findings are valuable for clinical practice, their generalizability should be interpreted with caution.

A third limitation concerns the methods used to diagnose cluttering. In the present study, cluttering was diagnosed using only MAR and RDF measures. Diagnosis of cluttering should be conducted from multiple perspectives (Van Zaalen-Op’t Hof and Reichel, 2015), and relying on only these two metrics may be insufficient. While one approach involves using a cluttering checklist, the accuracy of this method remains questionable (van Zaalen-Op’t Hof et al., 2009; Miyamoto, 2011; Tomisato et al., 2024a). Another approach depends on experts to make a diagnosis based on speech characteristics, but reports indicate that for this approach inter-rater agreement is not necessarily high (van Zaalen-Op’t Hof et al., 2009). For those reasons, we used MAR and RDF to diagnose cluttering. MAR and RDF have been previously demonstrated to have sufficient accuracy (Tomisato et al., 2024a). As this method is provisional, a more appropriate method needs to be developed. We are presently working on that.

A fourth limitation of the present study is that participants in the Cl-St-group were also evaluated using the stuttering assessment tools. The S-24-J, the UTBAS-6-A-J and the OASES-A-J questionnaires explicitly mention stuttering in their respective questions, it can be concluded that they are instruments designed specifically for the assessment of stuttering. Consequently, it can be inferred that these questionnaires may not be appropriate for individuals with cluttering. However, the participants in this study were individuals who presented with SDF as their chief complaint and in whom SDF was confirmed at a frequency of 1% or higher. Therefore, the use of questionnaires designed for stuttering on these participants with cluttering can be justified. The Impact Scale for Assessment of Cluttering and Stuttering (ISACS) is a questionnaire capable of simultaneously measuring the impact of both cluttering and stuttering (Kelkar et al., 2024). Although the ISACS is the most suitable questionnaire for this study, it could not be used because it was not yet available at the time of the present study’s design. Moreover, a Japanese version does not yet exist. There are very few tools available for measuring the effects of cluttering. This will present a significant challenge for clinicians and researchers in the future.

A fifth limitation is that SDF was used as an objective measure of the severity of disfluency. Other studies use %SS. Although the %SS and SDF are similar concepts, they are slightly different. The former does not categorize symptoms according to the “tension” of the disfluency, whereas in the latter, “tense word repetition” and “tense part word repetition” are considered, while “non-tense word repetition” and “non-tense part word repetition” are not considered as SDF (but rather as NDF). As with previous studies, the present study used SDF as a severity measure of disfluency to differentiate cluttering and stuttering (van Zaalen-Op’t Hof et al., 2009; Tomisato et al., 2024a) Notably, a previous study that used OASES scores to compare the severity of stuttering also used %SS (Blumgart et al., 2012). Both that study and ours demonstrated a significant correlation with scores on only Section 1 of the OASES, suggesting that %SS and SDF yield similar results. Although this is noted as a limitation due to differences in research methods, its impact on the conclusions of this study was minimal.

## 5 Conclusion

This study found that individuals who clutter and stutter, and those who only stutter, score similarly on standard tests that assess communication and anxiety. Considering the established co-occurrence of stuttering and SAD, these results suggest that the potential for comorbid SAD should be carefully considered in cases of cluttering with stuttering. Furthermore, the two groups exhibited distinct patterns in their psychological and cognitive profiles, including communication passivity, nonadaptive cognition, and impact on overall quality of life. Taken together, these preliminary findings substantiate the distinct natures of cluttering with stuttering versus ‘pure’ stuttering and underscore the need for a nuanced approach to their assessment and management.

## Data Availability

The raw data supporting the conclusions of this article will be made available by the authors, without undue reservation.
